# On the computational complexity of the maximum parsimony reconciliation problem in the duplication-loss-coalescence model

**DOI:** 10.1186/s13015-017-0098-8

**Published:** 2017-03-14

**Authors:** Daniel Bork, Ricson Cheng, Jincheng Wang, Jean Sung, Ran Libeskind-Hadas

**Affiliations:** 1 Department of Computer Science, Harvey Mudd College, Claremont, USA; 2 School of Medicine, University of Pittsburgh, Pittsburgh, USA; 3 School of Computer Science, Carnegie Mellon University, Pittsburgh, USA

**Keywords:** Phylogenetic reconciliation, Duplication-loss-coalescence model, NP-hardness, APX-hardness

## Abstract

**Background:**

Phylogenetic tree reconciliation is a widely-used method for inferring the evolutionary histories of genes and species. In the duplication-loss-coalescence (DLC) model, we seek a reconciliation that explains the incongruence between a gene and species tree using gene duplication, loss, and deep coalescence events. In the maximum parsimony framework, costs are associated with these event types and a reconciliation is sought that minimizes the total cost of the events required to map the gene tree onto the species tree.

**Results:**

We show that this problem is NP-hard even for the special case of minimizing the number of duplications. We then show that the problem is APX-hard when both duplications and losses are considered, implying that no polynomial-time approximation scheme can exist for the problem unless P = NP.

**Conclusions:**

These intractability results are likely to guide future research on algorithmic aspects of the DLC-reconciliation problem.

## Background

Phylogenetic tree reconciliation is a fundamental technique in the study of the evolutionary relationships of genes and species. Given a gene tree, a species tree, and the association between their leaves, we seek to explain the incongruence between the two trees using a set of evolutionary events. In the widely-used DL model, duplication and loss events are considered, while the DTL model considers horizontal transfers as well. Although these models can explain paralogous gene families, they do not address population effects. In contrast, coalescent models [1] can explain population effects but implicitly assume that all genes are orthologs. Recently, a new duplication-loss-coalescence (DLC) model has been proposed that combines the duplication-loss tree reconciliation model with a coalescent model. This DLC model has been shown to have higher accuracy of reconstructing evolutionary events than the DL model alone [2, 3].

Reconciliation is often performed using a maximum parsimony formulation in which each type of event in the model has an associated non-negative cost and the objective is to find a reconciliation of minimum total cost. Wu et al. [3] gave the first maximum parsimony algorithm for the DLC reconciliation problem. That algorithm has worst-case exponential time, leaving open the question of whether the problem can be solved in polynomial time.

In this paper, we show that the DLC parsimony problem is NP-hard and, further, has no polynomial-time approximation scheme (PTAS) unless P = NP. Specifically, we show that:

1. The DLC parsimony problem is NP-hard even when only seeking to minimize the number of duplications (i.e., loss and coalescence events have zero cost). However, the problem of minimizing duplications alone can be approximated using a PTAS for the multicut problem [4].
2. The DLC parsimony problem is APX-hard even when only duplications and losses are considered (i.e., coalescence events have zero cost), which implies that no PTAS exists for this problem unless P = NP.

Just as complexity results for DTL parsimony [5–7] guided the direction of algorithmic research on that problem, these results serve to guide future work on algorithms and heuristics for the DLC parsimony problem.

Finally, we note that while the DLC reconciliation problem considers duplications and losses, those events are treated differently from duplications and losses in the DL and DTL models due to the introduction of explicit gene loci in the DLC model. Thus, the complexity results that we offer here are not directly related to those for the DL and DTL models. The interested reader is referred to [5–7] for discussions of the DL and DTL models and known results about their computational complexity.

## Problem statement and preliminaries

This section provides notation, definitions, and basic results that will be used throughout the paper.

### Graph definitions

We begin with notation and definitions adapted from Wu et al. [3]. A *tree* is a rooted binary tree $T = (V(T), E(T))$ where *V*(*T*) denotes the set of nodes and *E*(*T*) denotes the set of directed edges, also called *branches*. An edge terminating at a leaf node is called a *terminal edge*. Denote by $L(T) \subset V(T)$ the set of leaves of *T*, $I(T) = V(T) \setminus L(T)$ the set of internal nodes, and $r(T) \in I(T)$ the root node. In a binary tree, leaves correspond to extant taxa whereas internal nodes correspond to ancestral taxa.

Denote by *c*(*v*) the set of children of *v*, *p*(*v*) the parent of *v*, and *e*(*v*) the directed edge (*p*(*v*), *v*). The partial orders $\le _T$ and $\ge _T$ on *V*(*T*) are defined by $u \le _T v$ if *u* is on the path from *r*(*T*) to *v* and $u \ge _T v$ if *v* is on the path from *r*(*T*) to *u*. Note that as required by a partial ordering, $\le _T$ and $\ge _T$ are reflexive ($u \le _T u$ and $u \ge _T u$). If $u \le _T v$ and $u \ne v$ then *u* is said to be an *ancestor* of *v* and *v* is a *descendant* of *u*. The *distance* from a node *u* to *v*, where $u \le _T v$, is the length of the shortest path from *u* to *v*. The *least common ancestor* of nodes *u*, *v*, denoted $lca (u, v)$, is the node *w* of maximum distance from *r*(*T*) such that $w \le _T u$ and $w \le _T v$. For two nodes $u, v \in T$, we say that an edge *e* *separates* *u* and *v* if *e* is either on the path from $lca (u,v)$ to *u* or on the path from $lca (u, v)$ to *v*. For convenience, we also use $lca$ as shorthand for the term “least common ancestor” when the context is clear.

### Reconciliations

A *leaf map* is a function $Le: L(G) \rightarrow L(S)$ that associates each leaf in the gene tree with the leaf in the species tree in which that gene is found. This function need not be one-to-one nor onto; gene tree leaves that map onto the same species tree leaf correspond to paralogous genes. The *labeled coalescent tree*, defined below, formalizes the notion of a reconciliation in the DLC model.

#### **Definition 1**

(*Labeled Coalescent Tree*) Given gene tree *G*, species tree *S*, and leaf map $$Le:L(G) \rightarrow L(S)$$, a *labeled coalescent tree (LCT)* for ($$G, S, Le)$$ is a tuple $$(\mathcal {M}, \mathbb {L}, \mathcal {L})$$ where:

- $\mathcal {M}: V(G) \rightarrow V(S)$ is a **species map** which maps each node of *G* to a node of *S*;
- $\mathbb {L}$ is a finite set, called the **locus set** of loci that have evolved within the gene family;
- $\mathcal {L}: V(G) \rightarrow \mathbb {L}$ is a **locus map** that maps each node of *G* to a locus in $\mathbb {L}$

subject to the following constraints:

1. If $$g \in L(G)$$, then $$\mathcal {M}(g) = Le(g)$$;
2. If $g \in I(G)$, then for $g' \in c(g)$, $\mathcal {M}(g) \le _S \mathcal {M}(g')$;
3. For $g, g' \in L(G)$ where $g \ne g'$, if $Le(g) = Le(g')$ then $\mathcal {L}(g) \ne \mathcal {L}(g')$;
4. For $\ell \in \mathbb {L}$, there exists $g \in V(G)$ s.t. $\mathcal {L}(g) = \ell$;
5. For $\ell \in \mathbb {L}$, let $N(\ell ) = \{g | g\in V(G); g \ne r(G); \mathcal {L}(g) = \ell ; \mathcal {L}(p(g)) \ne \ell \}.$ Then $|N(\ell )| \le 1$, where equality holds everywhere except for $\ell = \mathcal {L}(r(g))$.

Constraint 1 asserts that the species map $\mathcal {M}$ extends the leaf map *Le*; constraint 2 asserts that a gene node is mapped to either the same node or an ancestor of each of its children; constraint 3 asserts that since extant gene nodes (leaves) mapped to the same extant species (leaves) are paralogs, they must be mapped to different loci; constraint 4 asserts that the locus set only includes a locus if at least one gene uses that locus; and constraint 5 asserts that each locus is created only once.^1^

A gene node *g* is said to be a *speciation node* with respect to map $\mathcal {M}$ if for each child $g' \in c(g),$ $\mathcal {M}(g) \ne \mathcal {M}(g')$. Since a branch of the gene tree may span multiple branches of the species tree for a given map $\mathcal {M}$, *implied speciation nodes* are added as follows: For each non-root internal node $g \in I(G) \setminus \{ r(G) \}$ such that either (1) $p(\mathcal {M}(g)) \ne \mathcal {M}(p(g))$ or (2) *p*(*g*) is not a speciation node and $\mathcal {M}(g) \ne \mathcal {M}(p(g)),$ introduce a new node *h* and replace edge (*p*(*g*), *g*) with the pair of edges (*p*(*g*), *h*) and (*h*, *g*) and define $\mathcal {M}(h) = p(\mathcal {M}(g)).$ This process is repeated until there exists no node *g* that satisfies the conditions above. Wu et al. stipulate that the species map $\mathcal {M}$ is defined first, then implicit speciation nodes are added as required, and finally the locus map is defined on the vertices of the gene tree, which now includes any implied speciation nodes.

The set of gene nodes mapped to a given species node *s* is denoted $nodes (s) = \{g | g \in V(G); \mathcal {M}(g) = s\}$; $bottoms (s) = \{g | g \in nodes (s); g \in L(G) \vee \forall g' \in c(g), g' \notin nodes (s) \}$ is the subset of $nodes (s)$ whose children are mapped to descendants of *s*; and $tops (s) = bottoms (p(s))$.^2^ For any set $A \subset V(G)$, let $loci (A) = \{\ell | \exists g \in A \ \text{ s.t. } \ \ell = \mathcal {L}(g) \}$ denote the set of loci present on all genes in set *A*.

Next, Wu et al. define duplication and loss events. A duplication event corresponds to the creation of a new locus while a loss event corresponds to a locus that is present at either the top of a species branch, or created via a duplication within the species branch, but no longer present at the bottom of the species branch. More precisely, these events are defined as follows:

#### **Definition 2**

(*Duplication and Loss Events*) Let *G*, *S*, and $Le$ denote a gene tree, species tree, and leaf map $Le: L(G) \rightarrow L(S)$, respectively, and let ($\mathcal {M}$, $\mathbb {L}$, $\mathcal {L}$) be a LCT for $(G, S, Le)$.

- **Duplication events:** Let $g \in V(G)$, $g \ne r(G)$. If $\mathcal {L}(g) \ne \mathcal {L}(p(g))$ then *g* induces a *duplication event* on the edge $e(g) = (p(g), g)$.
- **Loss events:** Let $s \in V(S)$, $s \ne r(S)$. A locus $\ell \in \mathbb {L}$ induces a *loss event* on edge $e(s) = (p(s), s)$ if $\ell \in loci ( tops (s) \cup nodes (s)) \setminus loci ( bottoms (s))$.

**Fig. 1:**
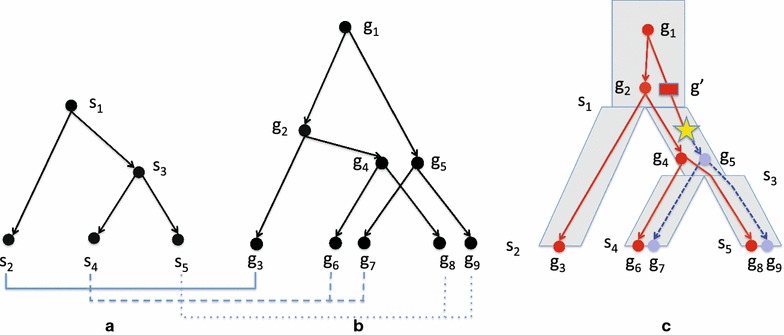
**a** A species tree and a** b** gene tree with the leaf map indicated by* solid*,* dashed*, and* dotted lines*.** c** A species and locus map for these two trees where circular nodes correspond to gene nodes in the gene tree and the rectangular node $g'$ is an implied speciation node. In this species map, $\mathcal {M}(g_1) = \mathcal {M}(g_2) = \mathcal {M}(g') = s_1$, $\mathcal {M}(g_3) = s_2$, $\mathcal {M}(g_4) = \mathcal {M}(g_5) = s_3$, $\mathcal {M}(g_6) = \mathcal {M}(g_7) = s_4$, and $\mathcal {M}(g_8) = \mathcal {M}(g_9) = s_5$. The two loci are indicated in* solid red* and* dashed blue*. There is a single duplication on edge $(g', g_5)$ indicated by a* star*. This edge separates paralogs $g_6$ and $g_7$ as well as paralogs $g_8$ and $g_9$

**Fig. 2:**
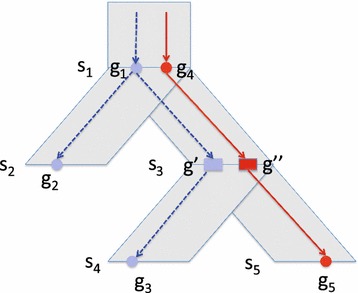
A part of a gene tree mapped onto a species tree.* Circular* nodes correspond to gene nodes in the gene tree and* rectangular* nodes $g'$ and $g''$ correspond to implied speciation nodes. The two loci are indicated in* solid red* and* dashed blue*. There is a loss on the edges $(g_1, g_2)$ and $(g', g_3)$ because the red locus is present at the tops of each of those edges but not at the bottoms of those edges. There is also a loss on edge $(g'', g_5)$ because the blue locus is present at the top of that edge but not the bottom

Figure 1 shows a (a) gene tree, (b) species tree, and (c) a species map and locus map with a duplication event. Figure 2 shows a subtree of a gene tree and a subtree of a species tree with the species and locus maps inducing multiple loss events.

#### **Definition 3**

(*Reconciliation Cost*) Given gene tree *G*, species tree *S*, leaf map $Le: L(G) \rightarrow L(S)$, and non-negative real number costs $C_d$ and $C_{\ell }$ for duplication and loss events, respectively, the *cost* of a LCT for $(G, S, Le)$ with *d* duplications events and $\ell$ loss events is $d C_d + \ell C_{\ell }$.

#### **Definition 4**

(*DCL Optimization Problem (DCLOP)*) Given gene tree *G*, species tree *S*, leaf map $Le: L(G) \rightarrow L(S)$, and non-negative costs $C_d$ and $C_{\ell }$ for duplication and loss events, find a LCT for $(G, S, Le)$ of minimum cost.

#### **Definition 5**

(*DCL Decision Problem (DCLDP)*) Given gene tree *G*, species tree *S*, leaf map $Le: L(G) \rightarrow L(S)$, non-negative costs $C_d$ and $C_{\ell }$ for duplication and loss events, and non-negative decision parameter *k*, does there exist a LCT for $(G, S, Le)$ of cost at most *k*?

### Duplication placement

Duplication events are determined entirely by the locus map $\mathcal {L}$ whereas loss events depend on both the species map and the locus map. For convenience in our subsequent analyses, we give an alternate characterization of the locus map and prove its equivalence with the original definition.

#### **Definition 6**

(*Duplication Placement*) Given gene tree *G*, species tree *S*, and leaf map $Le: L(G) \rightarrow L(S)$, a *duplication placement* is a subset *D* of the edges of *G* such that for every pair of leaves $g, g' \in L(G)$ where $g \ne g'$, if $Le(g) = Le(g')$ then *D* contains an edge that separates *g* and $g'$.

#### **Theorem 1**

*Given gene tree G, species tree S, and leaf map* $Le: L(G) \rightarrow L(S)$,* for every locus map* $\mathcal {L}$ *in a LCT inducing d duplication events, there exists a duplication placement D such that* $|D| = d$.* Conversely, for every duplication placement* *D* *such that* $|D| = d$,* there exists a locus map* $\mathcal {L}$ *that induces exactly d duplications.*

#### *Proof*

Let $\mathcal {L}$ be a locus map for $(G, S, Le)$ and define *D* to be the set of all edges $e(g) = (p(g), g)$, $g \in V(G)$, such that there is a duplication on edge *e*(*g*). By definition, |*D*| is the number of duplication events induced by $\mathcal {L}$. To show that *D* is a duplication placement, consider any pair of leaves $g, g' \in L(G)$ where $g \ne g'$ and $Le(g) = Le(g')$. By Definition 1 (3), $\mathcal {L}(g) \ne \mathcal {L}(g')$. Let *P* denote the path from $lca (g, g')$ to *g* and let $P'$ denote the path from $lca (g, g')$ to $g'$. There must exist some edge (*p*(*u*), *u*) in $P \cup P'$ such that $\mathcal {L}(u) \ne \mathcal {L}(p(u))$ since otherwise every node in *P* and $P'$ is mapped to the same locus, contradicting the assumption that $\mathcal {L}(g) \ne \mathcal {L}(g')$. Therefore, there is necessarily a duplication event on an edge in $P \cup P'$; this edges separates *g* and $g'$ and thus *D* is a duplication placement.

Conversely, let *D* be a duplication placement and consider the set $S(D) = \{G_1, \ldots , G_{|D|+1} \}$ comprising the $|D|+1$ subgraphs of *G* induced by the removal of the edges of *D* from *G*. Note that *S*(*D*) partitions the nodes *V*(*G*). Let $\mathbb {L}= \{1, \ldots , |D|+1 \}$ and let $\mathcal {L}$ map all nodes in $G_i$ to $i \in \mathbb {L}$. It follows directly that this satisfies the requirements of a locus map in Definition 1 (3), (4), (5). $\square$

Henceforth, we use locus maps and duplication placements interchangeably. When defining a duplication placement *D*, we say that a duplication is *placed* on an edge (*u*, *v*) to mean that edge (*u*, *v*) is included in the set *D*. We say that a duplication is *placed between* two leaves *g* and $g'$ to mean that there is a duplication placed on some edge that separates *g* and $g'$.

### 3SAT

Our reductions will be from 3SAT [8]: Given *m* Boolean variables $x_1, \ldots , x_m$ and *n* clauses $C_1, \ldots , C_n$ where each clause is the disjunction of exactly three literals over the given set of variables, we wish to determine whether there exists a valuation of the variables such that all clauses are satisfied. Without loss of generality, each literal occurs at most once per clause. In addition, the literals in the clauses are assumed to be ordered so that we may uniquely refer to the* h*th literal of each clause, $1 \le h \le 3$. Since the clauses are ordered, we may also uniquely refer to the* q*th occurrence of a literal $x_i$ (or $\overline{x}_i$) in the 3SAT instance. Finally, without loss of generality, we assume that no clause contains both a literal and its negation (since such clauses are trivially satisfied by every valuation and can thus be removed).

## NP-hardness

We show that DLCDP is NP-hard, even when loss events have cost zero, by a reduction from 3SAT. To provide intuition, we begin with a small example of the reduction and sketch the proof of correctness. Afterwards, we formalize the reduction and prove its correctness.

**Fig. 3:**
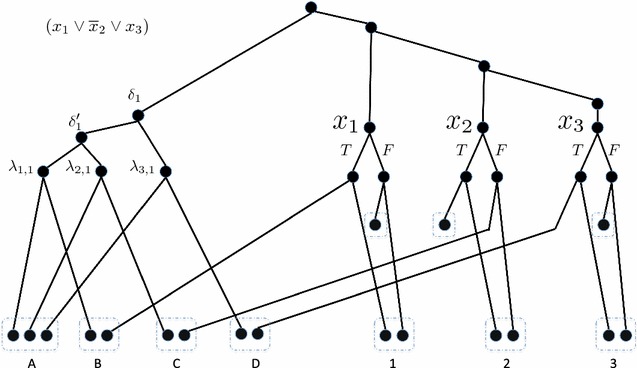
A small example of the reduction for a single clause $(x_1 \vee \overline{x}_2 \vee x_3)$

Figure 3 shows the construction for the 3SAT instance comprising a single clause, $(x_1 \vee \overline{x}_2 \vee x_3)$. We construct a gene tree with a single clause gadget on the left and one variable gadget for each of $x_1, x_2,$ and $x_3$ on the right. The variable gadget for variable $x_i$ is a subtree rooted at node $x_i$. That node has a true branch and a false branch, corresponding to setting that variable true or false, respectively. The nodes at the end of the true and false branches each have two children, resulting in four leaves for each variable gadget. (In general, variable gadgets can be larger than this, with size dependent on the number of clauses in which $x_i$ appears). One leaf on the true side and one leaf on the false side of the variable gadget get mapped to the same species tree leaf, as represented by the dashed rectangles at the bottom right of Fig. 3 and labeled 1, 2, and 3 for $x_1$, $x_2$, and $x_3$, respectively. Since each of these pairs of leaves are paralogs mapped to the same species leaf, there must be a duplication placed between them. As we shall see, our construction will force there to be a duplication on exactly one of the true or false branches incident on each $x_i$, corresponding to setting each variable to true or false, respectively.

Next, the gadget for clause 1 has five internal nodes (this is true in general, not just in this small example). The three important internal nodes are $\lambda _{1, 1}$, $\lambda _{2, 1}$, $\lambda _{3, 1}$ corresponding to the first, second, and third literals in clause 1. Each of these internal nodes has one leaf child that is mapped to a shared species leaf, as indicated in the dashed rectangle at the bottom left labeled *A*. Since the first literal in clause 1 is $x_1$, clause node $\lambda _{1, 1}$ and the true node in the variable gadget for $x_1$ each have a leaf child that is mapped to the same species node labeled *B* in the figure. Similarly, since the second literal of clause 1 is $\overline{x}_2$, clause node $\lambda _{2, 1}$ and the false node in the variable gadget for $x_2$ each have a leaf child that is mapped to the same species node labeled *C* in the figure. Finally, $\lambda _{3, 1}$ and the true node in the $x_3$ gadget have leaves on a shared species node *D*. All remaining leaves in the variable gadgets are mapped to their own individual unshared species leaves, placing no constraints on their locus mappings.

We set the cost of duplication events, $C_d$, to 1 and the cost of loss events, $C_{\ell }$, to 0. We set the decision parameter in this example to 5 which will force two duplications to be used in the clause gadget and one to be used in each of the three variable gadgets in a way that corresponds to choosing a valuation for the three variables (in general, the decision parameter for the number of duplications will be equal to the number of variables plus twice the number of clauses).

As noted earlier, the variable gadget leaves mapped to species 1, 2, and 3 require that there be at least one duplication placed within each variable gadget. Similarly, the three clause gadget leaves mapped to species *A* are paralogs and imply that there must be two duplications placed in the clause gadget rooted at $\delta _1$. Thus, in order to use no more than the five given duplications, there must be exactly one duplication placed in each variable gadget and exactly two duplications placed in the clause gadget. Moreover, without loss of generality, we can assume that duplications do not occur on edges terminating at leaves, since such duplications can be pushed up one level in the tree without violating any of the species map constraints.

We now sketch how the proof of correctness will proceed. First, assume that there is a satisfying assignment for the 3SAT instance (for example, $x_1 =$ true, $x_2 =$ true, $x_3 =$ false). We place duplications on the corresponding edges in the variable gadgets. This satisfies the requirement that there exists a duplication placed between each pair of leaves associated with species 1, 2, and 3. Since, in our valuation, $x_1 =$ true satisfies the clause, we choose *not* to place a duplication on the edge terminating at $\lambda _{1, 1}$ in the clause gadget, instead placing duplications on the two edges terminating at $\lambda _{2, 1}$ and $\lambda _{3, 1}$. This satisfies the requirement that a duplication is placed between each pair of the three clause leaves associated with species *A*. Moreover, the two leaves associated with species *B* have a duplication between them due to the duplication on $x_1$’s true edge and the leaves associated with groups *C* and *D* have duplications between them due to the duplications placed on the edges terminating at $\lambda _{2, 1}$ and $\lambda _{3, 1}$.

To prove the converse direction, we assume a solution to the constructed DLCDP instance; as noted above, this implies that there exists one duplication placed in each variable gadget and two in the clause gadget. At least one duplication must be placed in the subtree rooted at $\delta '_1$, as it is the $lca$ of two leaves in group *A*. Therefore, only one of the three remaining internal edges in the subtree rooted at $\delta _1$ can contain a duplication. Thus, at least one of the pairs of leaves mapped to species *B*, *C*, or *D* cannot be separated by a duplication placed inside the clause gadget and thus must be separated by a duplication placed inside a variable gadget. Consider, for example, the case that the pair of leaves in group *B* is separated by an edge in a variable gadget. By construction, that duplication must then occur on the true side of the $x_1$ gadget, which corresponds to setting $x_1$ to be true in the valuation which, in turn, satisfies this 3SAT instance.

### Formal reduction

Given a 3SAT instance with *m* variables $x_1,x_2,\ldots ,x_m$ and *n* clauses $C_1,C_2,\ldots ,C_n$, we construct an instance of DLCDP comprising *m* variable gadgets and *n* clause gadgets.

#### Variable gadgets

**Fig. 4:**
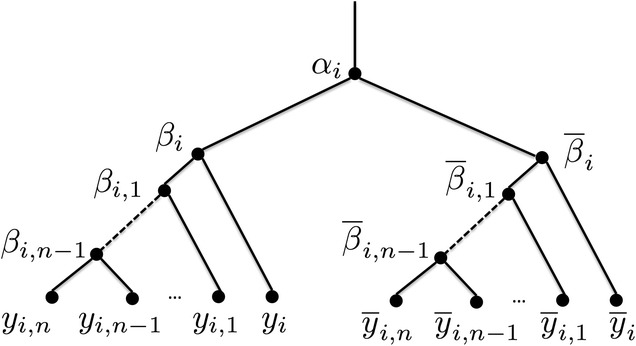
A variable gadget corresponding to variable $x_i$

A variable gadget for variable $x_i$, shown in Fig. 4, is a binary tree with root node $\alpha _i$ which, in turn, has two children $\beta _i$ and $\overline{\beta }_i$ which are roots of two subtrees. Node $\beta _i$ has two children: a leaf $y_i$ and an internal node $\beta _{i, 1}$. Each node $\beta _{i, k}$ has two children: a leaf $y_{i, k}$ and an internal node $\beta _{i, k+1}$, $1 \le k < n-1$. Node $\beta _{i, n-1}$ has two children: leaves $y_{i, n-1}$ and $y_{i, n}$. Similarly, node $\overline{\beta }_i$ has a child labeled $\overline{y}_i$ and another child $\overline{\beta }_{i, 1}$. Each node $\overline{\beta }_{i, k}$ has a child $\overline{y}_{i, k}$ and a child $\overline{\beta }_{i, k+1}$, $1 \le k < n-1$. Node $\overline{\beta }_{i, n-1}$ has children $\overline{y}_{i, n-1}$ and $\overline{y}_{i, n}$.

#### Clause gadgets

**Fig. 5:**
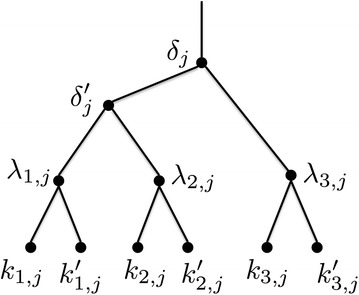
A clause gadget corresponding to clause $C_j$

A clause gadget for clause $C_j$, shown in Fig. 5, is a binary tree rooted at node $\delta _j$ which in turn has children $\delta '_j$ and $\lambda _{3, j}$. Node $\delta '_j$ has children $\lambda _{1, j}$ and $\lambda _{2, j}$. Finally, each node $\lambda _{h, j}$ has two leaf children, $k_{h, j}$ and $k'_{h, j}$, $1 \le h \le 3$.

#### Gene tree

The gene tree is constructed by assembling *m* variable gadgets and *n* clause gadgets into a single binary tree. Specifically, the gene tree is constructed from an arbitrary binary tree with $m+n$ leaves. The first *m* leaves become the roots of *m* variable gadgets corresponding to variables $x_1, \ldots , x_m$ while the remaining *n* leaves become the roots of *n* clause gadgets corresponding to clauses $C_1, \ldots , C_n$.

#### Species tree

The species tree is an arbitrary binary tree with $2mn + m + n$ leaves labeled $1, \ldots , 2mn + m + n$.

#### Leaf map

We define $Le: L(G) \rightarrow L(S)$ as follows:

1. $$Le(y_i)=Le(\overline{y}_i)=i$$, $$1 \le i \le m$$.
2. $$Le(k_{1,j})=Le(k_{2,j})=Le(k_{3,j})=m+j$$, $$1 \le j \le n$$,
3. For each $x_i$ appearing as the *h*-th literal in clause $C_j$, $Le(k'_{h,j})=Le(y_{i,j})=m+n+3(j-1)+h$,
4. For each $\overline{x}_i$ appearing as the *h*-th literal in clause $C_j$, $Le(k'_{h,j})=Le(\overline{y}_{i,j})=m+n+3(j-1)+h$,
5. Every leaf $g \in L(G)$ whose mapping is not specified above is mapped to a unique species leaf $s \in L(S)$ such no other gene leaf is mapped to *s*.

Note that steps 1 through 4 of this leaf map define the mapping of gene tree leaves onto species leaves $1, \ldots , m+4n$. By construction, after these first four steps in the leaf map, there remain $2mn - 3n$ gene tree leaves that are not yet mapped and $(2mn + m + n) - (m+4n) = 2mn - 3n$ species tree leaves that are not yet mapped onto. Thus, in step 5 of the leaf map, every gene tree leaf whose mapping was not established in parts 1 through 4 can be mapped to a unique species tree leaf.

#### Event costs and decision parameter

We set the cost of a duplication event to be 1 and all other event costs to be 0. The decision parameter is $2n+m$, meaning in this case that we seek a reconciliation with at most $2n + m$ duplications. It is easily seen that this reduction can be performed in time polynomial in the size of the given 3SAT instance.

### Proof of correctness

#### 3SAT $\rightarrow$ DLCDP

We first show that the existence of a satisfying valuation to a given 3SAT instance implies that the corresponding DLCDP instance is true. We prove this by constructing a duplication placement *D* of size $2n + m$ as follows: For each literal $x_i$, place a duplication on edge $e(\beta _i) = (\alpha _i,\beta _i)$ if $x_i$ is true in the valuation and place a duplication on edge $e(\overline{\beta }_i) = (\alpha _i,\overline{\beta }_i)$ if $x_i$ is false. This ensures that all pairs of leaves $y_i$ and $\overline{y}_i$, $1 \le i \le m$, are separated by an edge in *D* as required by part 1 of the leaf map above.

Next, consider an arbitrary clause $C_j$ and one of the literals $x_i$ whose true valuation satisfies $C_j$ (the case that the literal is $\overline{x}_i$ is analogous). Without loss of generality, assume that $x_i$ is the first literal in clause $C_j$ (the case that the literal is the second or third literal in the clause is analogous). The placement of a duplication on edge $e(\beta _i)$ ensures that leaves $k'_{1, j}$ and $y_{i, j}$ are separated by an edge in *D* as required by part 3 (analogously, part 4) of the leaf map. Next, we place duplications on the edges $e(\lambda _{2, j})$ and $e(\lambda _{3, j})$ in the clause gadget for $C_j$. This separates all leaves in part 2 of the leaf map and separates the remaining leaves in parts 3 and 4. Part 5 of the leaf map has no leaves requiring separation by *D*.

Since all of the duplication requirements implied by the leaf map are satisfied by this duplication placement and it uses exactly $k = 2n + m$ duplications, this is a solution to the constructed DLCDP instance.

#### DLCDP $\rightarrow$ 3SAT

Given a solution to the DLCDP instance, we construct a satisfying valuation for the corresponding 3SAT instance. Because part 1 of the leaf map associates each pair $y_i$ and $\overline{y}_i$, $1 \le i \le m$, with the same species node, each such pair must be separated by an edge in *D*. By construction, each such pair must be separated by a distinct edge in the variable gadget for $x_i$ which is either an edge on the path from $\alpha _i$ to $y_i$ or on the path from $\alpha _i$ to $\overline{y}_i$. Separating all such pairs therefore requires *m* edges in *D*.

For each clause $C_j$, $1 \le j \le n$, the leaves $k_{1,j}, k_{2,j}$, and $k_{3,j}$ are mapped to the same species leaf by part 2 of the leaf map. Therefore, each pair of those leaves must be separated by an edge in *D* and, by the construction of the clause gadget, this requires two edges in each clause gadget and thus a total of 2*n* additional edges in *D*.

Thus, all $k=2n+m$ are required to satisfy parts 1 and 2 of the leaf map, with exactly *m* edges selected from the variable gadgets and exactly 2*n* edges from the clause gadgets.

We construct a valuation of the boolean variables in the 3SAT instance as follows: for $1 \le i \le m$, set $x_i$ to true if there is a duplication placed on some edge on the path from $\alpha _i$ to $y_i$, and set $x_i$ to false if there is a duplication along the path from $\alpha _i$ to $\overline{y}_i$.

Consider an arbitrary clause $C_j$ and its corresponding gadget in the gene tree. Part 2 of the leaf map requires that there be an edge in *D* separating each pair of of $k_{1, j}$, $k_{2, j}$, and $k_{3, j}$, but, as noted above, only two edges of *D* are placed within that clause gadget. Since $\delta '_j$ is the lca of $k_{1, j}$ and $k_{2, j}$, at least one duplication must be placed in the subtree rooted at $\delta '_j$. Therefore, at least one of the three paths from $\delta _j$ to $k'_{1, j}$, $k'_{2, j}$, and $k'_{3, j}$ does not contain an edge in *D*. Without loss of generality, assume that the path from $\delta _j$ to $k'_{1, j}$ does not contain an edge in *D* and let $x_i$ be the first literal in clause $C_j$ (the argument is analogous if $x_i$ is the second or third literal of the clause). Then, by part 3 (analogously, part 4) of the leaf map, $k'_{1, j}$ and $y_{i, j}$ must be separated by an edge in *D*. Since this edge occurs in the variable gadget for $x_i$, by the observations above it must occur on the path from $\alpha _i$ to $y_i$, resulting in setting $x_i =$ true and thereby satisfying clause $C_j$.

Thus, all clauses are satisfied and the 3SAT instance is satisfiable. $\Box$

## APX-hardness of the DLC parsimony problem

When only the duplication cost is considered, the DLC optimization problem, DLCOP, can be approximated arbitrarily well using the polynomial-time approximation scheme (PTAS) for Multicut in binary trees [4] since duplications correspond exactly to removed edges in the Multicut problem. However, we now show that DLCOP has no PTAS in general, unless P = NP. Specifically, we show that DLCOP is APX-hard when duplications and losses are considered. We establish this result by a polynomial-time reduction from max3sat(b) which comprises a Boolean formula in 3-CNF form in which each variable appears at most *B* times in the clauses. Arora [9] showed that, for some $\epsilon$, $0< \epsilon < 1$, there exists a constant value of *B* ($B = 13$) and a polynomial-time reduction from any NP-complete problem $\Pi$ to max3sat(b) that maps *yes* instances of $\Pi$ to satisfiable instances of max3sat(b) and *no* instances of $\Pi$ to instances of max3sat(b) in which less than $1-\epsilon$ of the total number of clauses are satisfiable.

Our reduction maps an instance of max3sat(b) with *n* clauses (for sufficiently large values of *n*) to an instance of DLCOP and a parameter *b* such that the optimal solution to the DLCOP instance is less than *b* if the max3sat(b) instance is satisfiable and more than $(1+\alpha )b$ if at most $(1-\epsilon )n$ clauses can be satisfied, for some constant $\alpha >0$. If a polynomial-time $(1+\alpha )$-approximation algorithm exists for DLCOP, we can apply our gap-preserving reduction to generate a DLCOP instance from the max3sat(b) instance and then run the putative approximation algorithm to distinguish between satisfiable and $(1-\epsilon )$-satisfiable instances of max3sat(b). Thus, the existence of a $(1+\alpha )$-approximation algorithm for DLC implies that $P=NP$, and the approximation-hardness of DLCOP follows.

### Reduction

Given an instance of max3sat(b) comprising *m* variables and *n* clauses, we construct an instance of DLCOP comprising a gene tree, a species tree, a leaf map, and event costs. The reduction is based on the NP-hardness reduction in the previous section but introduces more complex gadgetry and uses nonzero cost for loss events.

#### Thorn gadget

**Fig. 6:**
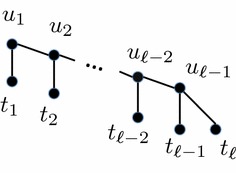
An $\ell$-thorn gadget

An $\ell$ *-thorn* gadget, depicted in Fig. 6, is a binary tree with $\ell$ leaves constructed as follows: let the root node be $u_1$. Each node $u_i$ has two children: internal node $u_{i+1}$ and leaf $t_i$, $1 \le i \le \ell -2$. Node $u_{\ell - 1}$ has two leaf children $t_{\ell -1}$ and $t_{\ell }$. Leaf $t_{\ell }$ is denoted the *end tip* of the thorn gadget.

#### Variable gadgets

**Fig. 7:**
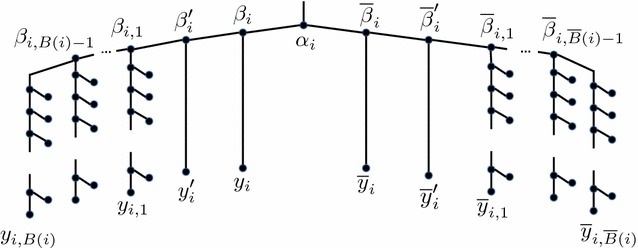
A gene tree variable gadget corresponding to variable $x_i$

Let *B*(*i*) and $\overline{B}(i)$ denote the number of occurrences of literals $x_i$ and $\overline{x}_i$, respectively. The variable gadget for variable $x_i$, illustrated in Fig. 7, consists of a root node, $\alpha _i$, and two subtrees, one for each of the two literals of this variable. The left subtree has root $\beta _i$, with two children: Internal node $\beta _i'$ and leaf $y_i$. In turn, $\beta _i'$ has two children: Internal node $\beta _{i,1}$ and leaf $y'_i$. Each node $\beta _{i, q}$, $1 \le q \le B(i)-2$, has a child $\beta _{i, q+1}$ and a second child which is the root of a $(n^2-1)$-thorn gadget with end tip $y_{i, q}$. Node $\beta _{i, B(i)-1}$ has two children, each of which is the root of a $(n^2-1)$-thorn gadget. The end tips of these thorn gadgets are labeled $y_{i, B(i)-1}$ and $y_{i, B(i)}$. This construction introduces a distinct $(n^2-1)$-thorn gadget for each occurrence of $x_i$ in the 3SAT instance. We refer to the thorn gadget terminating at end tip $y_{i, q}$ as *the thorn gadget for the* *q*th *occurrence of* $x_i$. The right subtree of $\alpha _i$, representing literal $\overline{x}_i$, is symmetric to the left subtree, but with $\beta _i$ and $\beta '_i$ replaced with $\overline{\beta }_i$ and $\overline{\beta }'_i$, respectively, each $\beta _{i, j}$ replaced by $\overline{\beta }'_{i, j}$, and each $y_{i, j}$ replaced by $\overline{y}_{i, j}$. This construction introduces a distinct $(n^2-1)$-thorn gadget for each clause containing $\overline{x}_i$. We refer to the thorn gadget terminating at end tip $\overline{y}_{i, q}$ as *the thorn gadget for the* *q*th *occurrence of* $\overline{x}_i$.

#### Clause gadgets

**Fig. 8:**
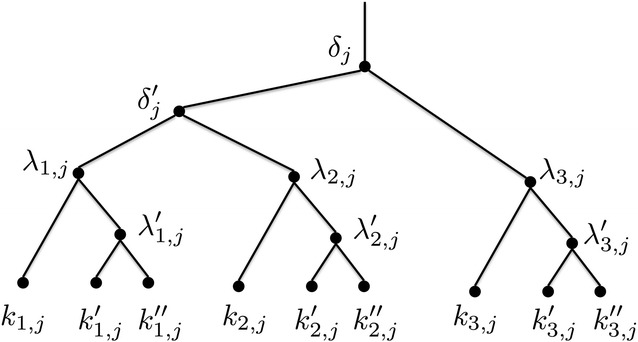
A gene tree clause gadget corresponding to clause $C_j$

A clause gadget corresponding to clause $C_j$, shown in Fig. 8, consists of root node $\delta _j$ with children $\delta '_j$ and $\lambda _{3, j}$. Node $\delta '_j$ has two children $\lambda _{1, j}$ and $\lambda _{2, j}$. Each node $\lambda _{h, j}$, $1 \le h \le 3$, is the root of a tree with two children, a leaf $k_{h, j}$ and a node $\lambda '_{h, j}$, which in turn has two leaf children $k'_{h, j}$ and $k''_{h, j}$.

#### Gene tree

The gene tree *G* is constructed as follows: the root of the gene tree is a node $g_0$ with children $g_1$ and $g_2$. Node $g_1$ is the root of a $(3n-m+1)$-thorn gadget. Node $g_2$ is the root of an arbitrary binary subtree with $n + m$ leaves. Each of the first *n* of those leaves becomes the root of a clause gadget for clauses $C_1, \ldots , C_n$ and the remaining *m* leaves become the roots of *m* variable gadgets for variables $x_1, \ldots , x_m$.

#### Species tree

**Fig. 9:**
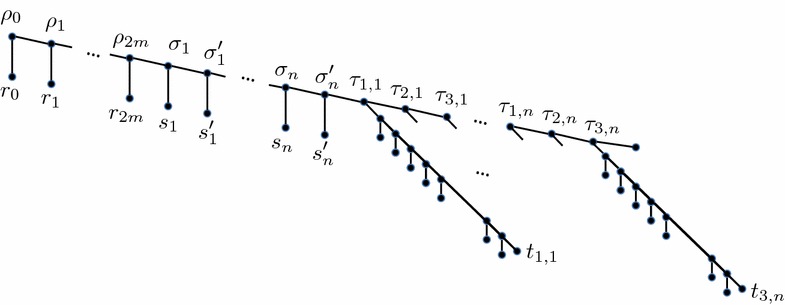
The species tree

The species tree, shown in Fig. 9, is rooted at node $\rho _0$ and is constructed from a path $\rho _0, \ldots , \rho _{2m}$ followed by $\sigma _1, \sigma '_1, \ldots , \sigma _n, \sigma '_n$, and finally $\tau _{1, 1}, \tau _{2, 1}, \tau _{3, 1}, \ldots , \tau _{1, n}, \tau _{2, n}, \tau _{3, n}$. This path is henceforth referred to as the *trunk* of the tree. Each node $\rho _i$ has a leaf child $r_i$, $1 \le i \le 2m$, and each node $\sigma _j$ and $\sigma '_j$ has a leaf child $s_j$ and $s'_j$, respectively, $1 \le j \le n$. Finally, each node $\tau _{h, j}$, which corresponds the* h*th literal in clause $C_j$, has a child that is the root of a $n^2$-thorn with end tip $t_{h,j}$ (henceforth referred to as *the* $n^2$ *-thorn for* $\tau _{h, j}$), $1 \le h \le 3$, $1 \le j \le n$. Node $\tau _{3, n}$ has an additional leaf child so that the tree is binary.

#### Leaf map and event costs

The leaf map *Le* is defined as follows:

1. $$Le(y_i)=Le(\overline{y}_i)=r_{2i-1}$$ and $$Le(y_i') = Le(\overline{y}_i') = r_{2i}$$, $$1 \le i \le m$$;
2. $$Le(k_{1,j})=Le(k_{2,j})=Le(k_{3,j})=s_j$$ and $$Le(k_{1,j}')=Le(k_{2,j}')=Le(k_{3,j}')=s'_{j}$$, $$1 \le j \le n$$;
3. Each leaf in the $(3n-m+1)$-thorn gadget rooted at node $g_1$ is mapped to $r_0$;
4. If the* h*th literal of $C_j$ is $x_i$ and this is the* q*th occurrence of $x_i$ in the 3SAT instance, then each leaf of the $(n^2-1)$-thorn gadget for the* q*th occurrence of $x_i$ is mapped to the leaf with the same index in the $n^2$-thorn gadget for $\tau _{h, j}$ and $k''_{h, j}$ is mapped to the end tip, $t_{h, j}$, of that $n^2$-thorn gadget.
5. If the* h*th literal of $C_j$ is $\overline{x}_i$ and this is the* q*th occurrence of $\overline{x}_i$ in the 3SAT instance, then each leaf of the $(n^2-1)$-thorn gadget for the* q*th occurrence of $\overline{x}_i$ is mapped to the leaf with the same index in the $n^2$-thorn gadget for $\tau _{h, j}$ and $k''_{h, j}$ is mapped to the end tip, $t_{h, j}$, of that $n^2$-thorn gadget.

Let the event costs be as follows: $D=2Bn^2, L=1, C=0$. Finally, note that this reduction can be performed in polynomial time.

### Proof of correctness

To prove the correctness of our reduction, we show that:

- If the max3sat(b) instance is satisfiable, the optimal cost of the constructed DLC instance is less than
  $$\begin{aligned} b=(10B + 2)n^3 + 121 n^2 \end{aligned}$$
- For sufficiently large *n*, if at most $(1-\epsilon )n$ clauses of the max3sat(b) instance can be satisfied, the optimal cost is more than $(1+\alpha )b$, where
  $$\begin{aligned} \alpha =\frac{\epsilon }{20B+4} \end{aligned}$$

#### Satisfiable MAX3SAT(B) instances

We first consider a satisfiable instance of max3sat(b). We show how a satisfying valuation can be used to construct a solution to the DLC instance whose cost is less than *b*.

The species map $\mathcal {M}$ maps all internal nodes of *G* to $\rho _0$ except for $g_1$ and its descendant $(3n-m+1)$-thorn gadget which are mapped to $r_0$; each leaf $g \in L(G)$ is mapped to $Le(g)$.

For each variable $x_i$, we place one duplication in the corresponding variable gadget, on the edge $e(\overline{\beta }_i)$ if $x_i$ is assigned true and on the edge $e(\beta _i)$ if $x_i$ is assigned false.^3^ This ensures that $y_i$ and $\overline{y}_i$ are separated and that $y'_i$ and $\overline{y}'_i$ are separated, as required by part 1 of the leaf map. For each clause $C_j$, identify any one literal that satisfies that clause. If the first literal in $C_j$ satisfies the clause, place duplications on edges $e(\lambda _{2, j})$ and $e(\lambda _{3, j})$. Alternatively, if the second literal in $C_j$ satisfies the clause, place duplications on edges $e(\lambda _{1, j})$ and $e(\lambda _{3, j})$; alternatively, if the third literal in $C_j$ satisfies the clause, place duplications on edges $e(\lambda _{1, j})$ and $e(\lambda _{2, j})$. This placement of two duplications per clause gadget satisfies the constraints implied by part 2 of the leaf map, which requires that each pair of $k_{1,j}, k_{2,j}, k_{3,j}$ be separated and that each pair of $k'_{1,j}, k'_{2,j}, k'_{3,j}$ be separated. Thus far, $m+2n$ duplications have been placed. Finally, we place $3n-m$ duplications on the terminal edges of the $(3n-m+1)$-thorn gadget, since all $3n-m+1$ of its leaves are mapped to $r_0$ by part 3 of the leaf map and thus each pair of leaves must be separated. Note that parts 4 and 5 of the leaf mapping do not map multiple species leaves to the same trees leaves and thus require no additional duplication placements. The total number of duplications is thus $m+2n+(3n-m)=5n$.

Next, we count the number of losses. We do this by first counting losses on the $n^2$-thorns of the species tree and then on the trunk of the species tree.

Each clause $C_j$ has three $n^2$-thorns in the species tree, one branching from each of $\tau _{1, j}$, $\tau _{2, j}$, and $\tau _{3, j}$. Without loss of generality, assume that clause $C_j$ is satisfied by its first literal and thus duplications were placed on $e(\lambda _{2, j})$ and $e(\lambda _{3, j})$. Also, without loss of generality, assume that the first literal in $C_j$ is $x_i$ (the case for $\overline{x}_i$ is analogous) and that this is the $q$th occurrence of $x_i$ in the 3SAT instance. The duplication on $e(\lambda _{2, j})$ implies that leaf $k''_{2, j}$ is mapped to a different locus than all of the leaves of the $(n^2-1)$-thorn for the $q$th occurrence of $x_i$ in the variable gadget for $x_i$. Since $Le(k''_{2, j}) = t_{2, j}$ by part 4 of the leaf map, there is a loss event on each of the $n^2$ edges terminating at the leaves of the $n^2$-thorn gadget for $\tau _{2, j}$. Similarly, the duplication on edge $e(\lambda _{3, j})$ incurs $n^2$ losses in the $n^2$-thorn gadget for $\tau _{3, j}$ for a total of $2n^2$ losses for clause $C_j$. Since $C_j$ is satisfied by $x_i$, we know that $x_i =$ true and thus a duplication was placed on edge $e(\overline{\beta }_i)$ in the variable gadget for $x_i$. Therefore, there is no duplication placed between $k''_{1, j}$ and the leaves of the $(n^{2}-1)$-thorn for the $q$th occurrence of $x_i$ and thus there are no losses incurred on the $n^2$-thorn for $\tau _{1, j}$. Since there are *n* clauses and each contributes $2n^2$ losses in the corresponding $n^2$-thorns, $2n^3$ losses are incurred thus far.

We next consider the number of losses incurred on the trunk of the species tree. Since $\mathcal {M}(g_1) = r_0$, none of the loci created by the $3n-m$ duplications in the $3n-m+1$-thorn required by part 3 of the leaf map induce loss events. There are $1+2m+2n+3n$ nodes on the trunk and at most $m+2n$ loci can be lost on each of the two edges emanating from each such node since there only $m+2n$ other duplications.

Observing that $m \le 3n$, the total number of losses can thus be bounded from above by

$$\begin{aligned} 2(m+2n)(1+2m+2n+3n)&\le 2\cdot 5n \cdot 12n <121n^2. \end{aligned}$$

Therefore, the total cost of this solution is bounded by

$$\begin{aligned} 5n\cdot 2Bn^2 + (2n^3+121n^2)\cdot 1 = (10B+2)n^3+121n^2 =b. \end{aligned}$$

#### At most (1-$\epsilon$)-satisfiable MAX3SAT(B) instances

To complete the proof, we show that given an instance of max3sat(b) in which the fraction of satisfiable clauses is at most (1-$\epsilon$), the optimal cost of the corresponding DLC instance, for sufficiently large *n*, is greater than:

$$\begin{aligned} (1+\alpha )b&= \left( 1+\frac{\epsilon }{20B+4} \right) \left( (10B+2)n^3+121n^2 \right) \\&= (10B+2)n^3 + \frac{\epsilon }{20B+4}(10B+2)n^3 + \left( 1+\frac{\epsilon }{20B+4} \right) 121n^2 \\&= (10B+2)n^3 + \frac{\epsilon }{2} n^3 + \left( 1+\frac{\epsilon }{20B+4} \right) 121n^2 \\&=\left( 10B+2+\frac{\epsilon }{2} \right) n^3+\left( 1+\frac{\epsilon }{20B+4} \right) 121n^2. \end{aligned}$$

Part 1 of the leaf map requires at least one duplication placement per variable gadget, part 2 of the leaf map requires at least two duplications per clause gadget, and part 3 of the leaf map requires $3n-m$ duplications to be placed in the $(3n-m+1)$-thorn gadget. Therefore, all valid duplication placements for this instance use at least $m + 2n + (3n-m) = 5n$ duplications. We call a solution that uses exactly 5*n* duplications *well-behaved*.

A *well-behaved* solution must use exactly one duplication in each variable gadget. For each variable gadget for variable $x_i$, this duplication must be placed on either the edge $e(\beta _i)$ or the edge $e(\overline{\beta }_i)$ in order to separate both $y_i$ and $\overline{y}_i$ and $y'_i$ and $\overline{y'}_i$. We interpret a duplication on edge $e(\beta _i)$ as setting variable $x_i$ to false and a duplication on edge $e(\overline{\beta }_i)$ as setting $x_i$ to true. Thus, a well-behaved solution to the DLC Optimization Problem has a corresponding valuation of the variables in the 3SAT instance.

We now show that all optimal solutions to the DLC Optimization Problem are necessarily well-behaved. Consider a solution for our constructed DLC instance that is not well-behaved and thus comprises more than 5*n* duplications. A duplication placed outside of a variable, clause, or $(3n-m+1)$-thorn gadget cannot satisfy any of the duplication requirements imposed by the leaf map and thus can be removed, reducing the number of duplications and not increasing the number of losses.

If a variable gadget for $x_i$ contains more than one duplication, we may replace all duplications in that variable gadget with a single duplication on edge $e(\beta _i) = (\alpha _i, \beta _i)$, which satisfies the duplication requirements of the leaf map and reduces the number of duplications by at least one. Introducing a new duplication may increase the number of losses. However, since each variable $x_i$ appears in at most *B* clauses in the max3sat(b) instance, the number of new losses introduced can be at most $Bn^2$ due to the *B* $n^2$-thorn gadgets where losses are introduced and the *O*(*n*) internal vertices in the trunk of the species tree, which is dominated by $Bn^2$ for sufficiently large *n*. Thus, the total number of new losses incurred is less than $2Bn^2$ for sufficiently large *n* and thus less than the cost of the saved duplication.

Similarly, if a clause gadget for $C_j$ contains more than two duplications, we can replace it with two duplications on the edges $e(\lambda _{1,j})$ and $e(\lambda _{2,j})$. The saving of one duplication is larger than the cost of the additional losses.

We have established that an optimal solution to the constructed DLC instance is necessarily well-behaved. Next, observe that any species map must map $\lambda '_{h, j}$, $1 \le h \le 3$, $1 \le j \le n$, to a node *v* on the trunk of the species tree such that $v \le _T \tau _{h, j}$ since $\lambda '_{h, j}$ has children $k'_{h, j}$ and $k''_{h, j}$ and $Le(k'_{h, j}) = s'_j$ while $Le(k''_{h, j}) = t_{h, j}$.

Consider an optimal solution for the DLC instance. Since this solution is well-behaved, it induces a valuation of the Boolean variables as described above. As noted earlier, if clause $C_j$ is satisfied by this valuation then a total of $2n^2$ losses are incurred in two of the three $n^2$-thorns $\tau _{1, j}$, $\tau _{2, j}$, and $\tau _{3, j}$. Conversely, if clause $C_j$ is not satisfied by this valuation then a total of $3n^2$ losses are incurred in all three of those $n^2$-thorns. To see this, let the $h$th literal, $1 \le h \le 3$, of $C_j$ be $x_i$ (analogously, $\overline{x}_i$) and let this be the $q$th occurrence of this literal in the 3SAT instance. Since $C_j$ is not satisfied $x_i =$ false [analogously, $\overline{x}_i =$ false and, therefore, there is a duplication placed on edge $e(\beta _i)$ (analogously, $e(\overline{\beta }_i)$]. It follows that the loci of the leaves of the $(n^{2}-1)$-thorn for the $q$th occurrence of $x_i$ are different from the locus of $k''_{h, j}$, causing $n^2$ losses in the $n^2$-thorn for $\tau _{h, j}$ since, as noted above, the path from $\mathcal {M}(\lambda '_{h, j})$ to $\mathcal {M}(k''_{h, j}) = t_{h, j}$ passes through every internal node of this thorn gadget. Thus, if $C_j$ is unsatisfied, its three $n^2$-thorns in the species tree contribute $3n^2$ losses.

We have shown that every satisfied clause contributes $2n^2$ losses and every unsatisfied clause contributes $3n^2$ losses. Therefore, if there are fewer than $2n^3 + \epsilon n^3$ losses then there must be fewer than $\epsilon n$ unsatisfied clauses. Since there are more than $\epsilon n$ unsatisfied clauses by assumption, for sufficiently large *n*, the cost of a well-behaved solution, and thus of an optimal solution, is at least:

$$\begin{aligned} 5n(2Bn^2) + 2n^3 + \epsilon n^3&= (10B+2+\epsilon )n^3 \\&> \left( 10B+2+\frac{\epsilon }{2} \right) n^3+\left( 1+\frac{\epsilon }{20B+4} \right) 121n^2\\&= (1+\alpha )b \end{aligned}$$

$$\Box$$

## Conclusion

We have shown that the DLC parsimony problem is NP-hard even when only duplications are considered and APX-hard when duplications and losses are considered. These results may help guide the direction of future research on algorithms and heuristics for the DLC parsimony problem. In particular, although the existence of a polynomial-time approximation scheme for the DLC parsimony problem would imply that P = NP, approximation algorithms may exist and would be of significant potential value.

## Footnotes

1 Wu et al. include one other relation in the LCT, a partial ordering relation $\mathcal {O}$ on *V*(*G*). This relation is used solely to define and count the number of so-called *coalescence events*. In this paper, we show that the reconciliation problem is intractable even when the cost of coalescence events is ignored. Therefore, while coalescence events are induced by the LCT, there is no need to explicitly identify these events here and we thus omit the partial ordering relation $\mathcal {O}$ from the above definition.

2 Wu et. al use the names *leaves* and *roots* where we use *bottoms* and *tops*, respectively.

3 Note that this is opposite of what was done in the preceeding NP-hardness proof. This switch is necessary as becomes evident in the subsequent proof of correctness.
